# Sensitive terahertz plasmonic metasurface biosensor integrated with microfluidics†

**DOI:** 10.1039/d5na00312a

**Published:** 2025-06-12

**Authors:** Amir MoradiFotouhi, Mahdi Pourfath

**Affiliations:** a School of Electrical and Computer Engineering, University of Tehran Tehran Iran; b Institute for Microelectronics, TU Wien Gusshausstrasse 27-29/E360 1040 Vienna Austria pourfath@ut.ac.ir pourfath@iue.tuwien.ac.at

## Abstract

In recent years, substantial research has driven the development of low-cost biosensors in the terahertz (THz) range. This study introduces an advanced biosensor structure that integrates a monolayer graphene strip and a gold bar within a microfluidic channel, specifically optimized to reduce environmental impact and enhance sensitivity in biological detection. The unique design incorporates a tunable plasmon-induced transparency (PIT) mechanism, enabling precise control of the coupling between dark and bright modes (graphene and gold) to achieve high sensitivity. To analyze this structure comprehensively, Maxwell's equations were solved using the finite element method (FEM) to extract *S*-parameters, while the Lorentz oscillator model was employed to verify the damping rates and coupling coefficient of the PIT effect. Furthermore, the sensor's sensitivity can be finely adjusted by modifying its geometric parameters during fabrication and by applying an electric field. By correlating PIT resonance shifts with analyte variations within the channel, this biosensor demonstrates a sensitivity of approximately 700 GHz per RIU, highlighting its significant potential in THz biosensing applications.

## Abstract 2

The hybrid platform enables label-free detection of cancer and infectious disease biomarkers through sharp plasmonic resonance shifts, offering a promising route for rapid and highly sensitive biomedical diagnostics.

## Graphical abstract

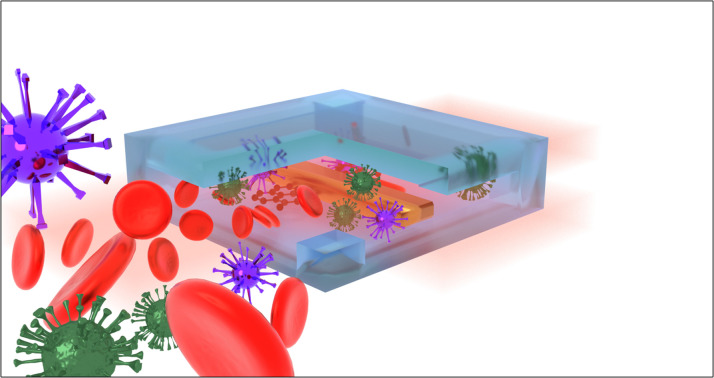

## Introduction

Metasurfaces, comprised of two-dimensional (2D) arrays of light scatterers combining metal, dielectric, and 2D materials,^1^ have garnered extensive attention due to their ability to significantly alter the phase, amplitude, and polarization of incident free-space light beams through sub-wavelength separated nano-resonators.^2^ The interaction between light and these scatterers can be precisely controlled by adjusting the nano-antennas’ geometrical parameters, facilitating the tuning of the metasurfaces' optical properties.

Recent advancements have seen the incorporation of phase-change materials such as vanadium dioxide (VO_2_),^3^ Ge_2_Sb_2_Te_5_,^4,5^ and graphene^6^ into metasurfaces, enabling dynamic control of their properties in response to external stimuli.

Graphene, characterized by its unique electronic, optical, and plasmonic properties,^7^ presents a versatile platform for the rapid and dynamic manipulation of light.^8^ The capabilities of graphene plasmons extend to facilitating strong light–matter interactions^9^ and enabling the tuning of electronic and optical properties through methods such as electrostatic gating,^10,11^ chemical doping,^12^ and the application of strain.^13^ Given the natural vibration frequencies of biomolecules predominantly lie in the THz region,^14^ detectors operating within this spectrum are particularly advantageous for biosensing applications.^15^ The responsiveness of graphene plasmons in the THz range makes graphene an ideal candidate for detecting biological species, a capability unattainable with traditional metallic plasmonic materials.^16^ To benefit from strong plasmonic properties of metallic nanostructures and tunable graphene plasmons in the THz regime,^17,18^ one can integrate both to achieve hybrid metal–graphene plasmons, offering enhanced performance in plasmonic applications.^12^

The concept of the PIT,^19,20^ leveraging active metal–graphene plasmon polaritons, mirrors the phenomena of electromagnetically induced transparency (EIT) observed in atomic systems.^21^ Unlike EIT, which is constrained by the requirement for low operational temperatures,^22^ PIT can be employed to achieve ultra-sensitive bio-sensors.^23^ This emergent property originates from the constructive interference between two distinct modes – dark and bright^15^ – which can be excited through near-field coupling between the two modes.^24^

The exploitation of PIT for the development of ultra-sensitive biosensors, among other applications, underscores its potential in revolutionizing the field of plasmonics^25–27^ and biosensing.^28–32^ However, the performance of THz biosensors can be compromised by factors such as the reduced volume of liquid samples^15^ and environmental influences,^31^ highlighting the importance of integrating microfluidic technologies.^33^

The reduction in liquid-testing samples refers to the decreased volume of analyte required for testing, which enhances the sensitivity and accuracy of the biosensor.^14^ Environmental influences include temperature fluctuations, humidity, and external vibrations, all of which can affect the performance of the biosensor. Shifts in refractive index serve as the primary indicator of these changes, allowing the biosensor to detect even minute variations in the surrounding environment. These technologies not only mitigate the impact of environmental factors but also enhance the precision and efficiency of biosensors, as demonstrated by their application in detecting liver cancer biomarkers.^15^

In response to these challenges, this work introduces a tunable PIT-based biosensor that synergizes the plasmonic properties of gold and graphene within a microfluidic environment, optimizing both static and dynamic tunability while safeguarding against environmental variabilities. The proposed structure opens avenues for breakthroughs in biosensing technology, providing increased sensitivity and adaptability in identifying a diverse array of biological markers.

## The structure geometry

The proposed sensor consists of a multilayer design optimized for high-performance terahertz biosensing. A gold resonator on the top layer supports the excitation of bright plasmonic modes, ensuring strong interaction with incident THz radiation. Beneath it, a graphene monolayer enables tunable dark modes through electrostatic gating, allowing dynamic control of the PIT effect. Finally, a SiO_2_ layer serves as both the dielectric substrate and the microfluidic channel, offering chemical stability and low THz absorption. This hybrid configuration combines the strengths of each material—resonance strength, tunability, and environmental robustness—to enhance sensing performance and spectral control.

**Fig. 1:**
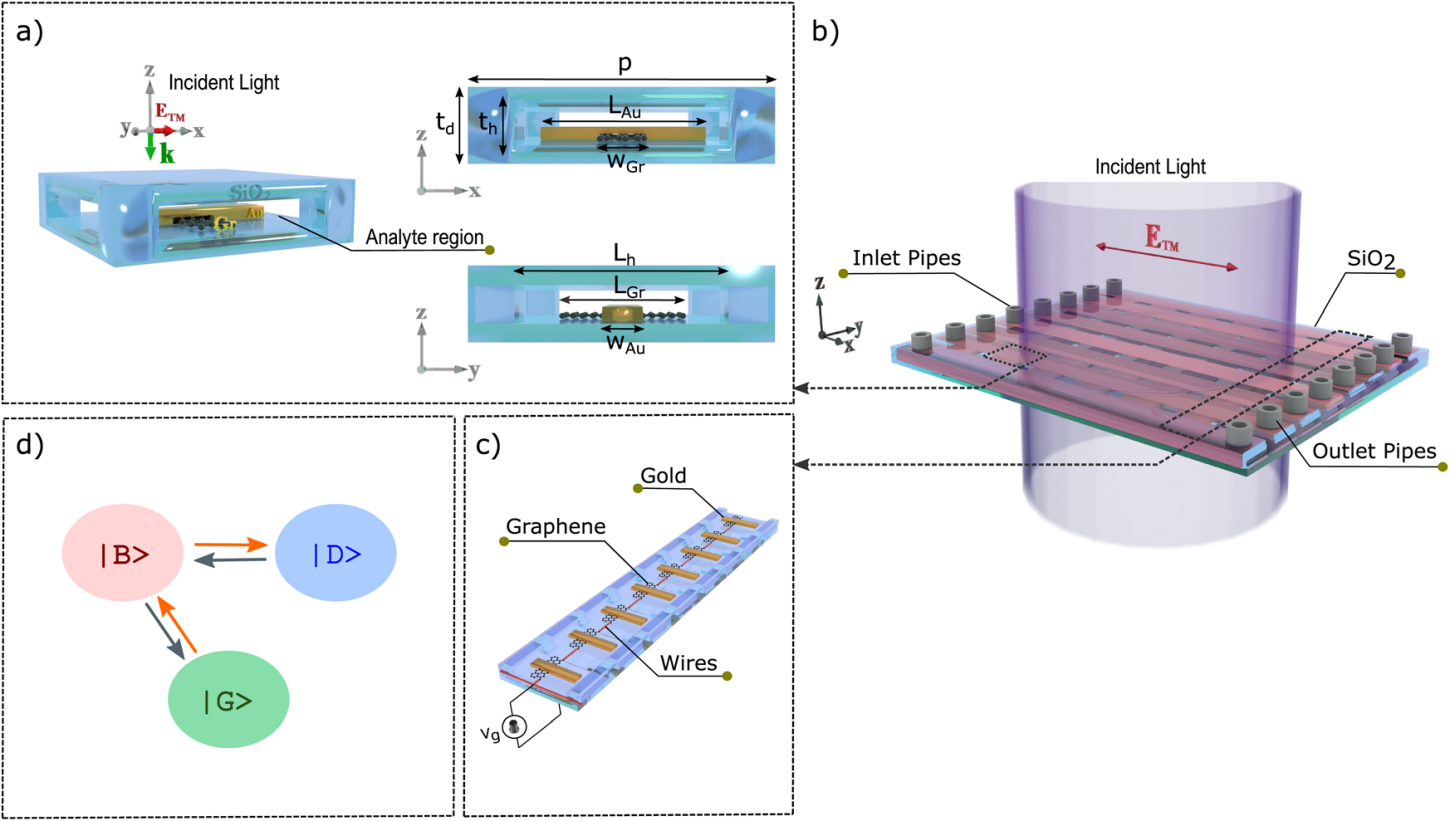
Schematic illustration of the structure based on glod bars and graphene strips (a) graphene strip and gold bar were periodically distributed along *y*-axis and *x*-axis directions into the SiO_2_ dielectric. The cross-sections of a unit cell along *x*–*z* and *y*–*z* are shown with these parameters: *t*_Au_ = 200 nm, *W*_Au_ = 12 μm, *L*_Au_ = 50 μm, *L*_Gr_ = 35 μm, *W*_Gr_ = 6 μm, *L*_h_ = 44 μm, *t*_h_ = 10 μm, and *p* = 60 μm. (b) The 3D-model of the device structure where the silicon inlet and outlet pipes are placed on both sides of the structure to fill and empty the analytes. (c) Applying a bias voltage to the graphene strips by gold wires. (d) States of the PIT phenomenon are created by the dark and bright modes under *x*-polarized incident light.

Fig. 1(a) demonstrates the schematic of the proposed unit cell including a graphene strip, a gold bar, and silicon dioxide SiO_2_ used for the pipe with its low-index and low THz-wave absorption. The graphene and gold were embedded into the pipe with *t*_d_ = 16 μm thickness. The dimensions of the gold and graphene are *t*_Au_ = 200 nm, *W*_Au_ = 12 μm, *L*_Au_ = 50 μm, *L*_Gr_ = 35 μm, and *W*_Gr_ = 6 μm which the gold bars are periodically distributed along the *x*-direction and graphene strips are perpendicularly deposited under them. The structure is periodic with a periodicity of *p* = 60 μm along the *x*- and *y*-directions. The geometrical parameters for perpendicular pipes are *L*_h_ = 44 μm, and *t*_h_ = 10 μm. Fig. 1(b) shows the structure that consists of inlet and outlet pipes that are designed to inject and vacate analyte, graphene strips and gold bars are embedded in the glass dielectric that can be excited by applying an *x*-polarized transverse magnetic wave 
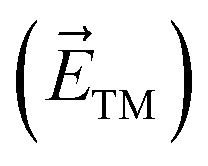
 along the *z*-direction.

The fabrication of the proposed THz biosensor begins with a high-resistivity silicon dioxide (SiO_2_) substrate, which is thoroughly cleaned using standard semiconductor cleaning techniques such as RCA or piranha cleaning to remove organic and particulate contaminants, as described by Kern.^34^ A positive photoresist is spin-coated onto the substrate and patterned using UV lithography to define the microchannel layout. Reactive ion etching (RIE) with CHF_3_/O_2_ plasma chemistry is subsequently employed to etch into the SiO_2_, forming a microchannel with well-controlled depth and vertical sidewalls, following the method outlined by Gottscho *et al.*^35^ After etching, the residual photoresist is stripped off, yielding a clean microchannel structure. Next, a monolayer graphene film synthesized *via* chemical vapor deposition (CVD) on a copper foil is transferred onto the floor of the etched microchannel, using the PMMA-supported wet-transfer process proposed by Lee *et al.*^36^ A thin layer of poly(methyl methacrylate) (PMMA) is first spin-coated onto the graphene/copper foil and baked to provide mechanical support. The underlying copper is removed by immersion in an aqueous etchant such as ammonium persulfate. After thorough rinsing in deionized water, the PMMA/graphene stack is carefully transferred onto the SiO_2_ substrate. Following drying, the PMMA layer is dissolved in acetone, leaving a clean, continuous graphene monolayer conformally attached to the microchannel floor. Subsequently, electron-beam lithography (EBL) is utilized to define the gold nanoantenna structures. A thin PMMA resist layer is spun onto the graphene and patterned by high-resolution e-beam exposure. Following development, a Ti/Au (5 nm/45 nm) bilayer is deposited using electron-beam evaporation, and lift-off is performed to leave well-defined gold nanoantennas directly on the graphene sheet, as demonstrated by Bae *et al.*^37^ Finally, the microchannel is sealed by bonding an unpatterned flat SiO_2_ cover onto the structured substrate. No plasma activation is used; instead, the two surfaces are physically aligned and joined primarily through van der Waals interactions. To improve bonding quality, a mild thermal treatment at approximately 100 °C is optionally applied, following the approach of Park *et al.*^38^ The final structure consists of a hermetically sealed microchannel lined with a graphene–gold hybrid surface, specifically designed for high-sensitivity THz biosensing applications without requiring any additional surface functionalization.^39^

The Fermi level of graphene can be adjusted by the gate bias voltage through gold wires as shown in Fig. 1(c).^40^ The PIT system includes a radiative (bright mode) |*B*〉 and a dark |*D*〉 state with the same resonant frequency of *ω*_0_, see Fig. 1(d).^41^ A graphene as a dark mode |*D*〉 can not be directly excited with the incident wave, thus a transition between |*G*〉 and |*D*〉 is forbidden. The coupling between two modes can be tuned by changing the position of the graphene under the gold that results in another light excitation pathway |*G*〉 → |*B*〉 → |*D*〉 → |*B*〉. Therefore, two excitation pathways |*G*〉 → |*B*〉 and |*G*〉 → |*B*〉 → |*D*〉 → |*B*〉 are possible.^42^

## Results and discussion

The proposed device structure can be tuned in two ways. Static tunability can be achieved by adjusting the dimensions of the device components during fabrication, such as the glass channels, the gold bars, and graphene strips, as well as their relative positions to each other. Dynamic tunability can be obtained by controlling the Fermi level of the graphene strips.

**Fig. 2:**
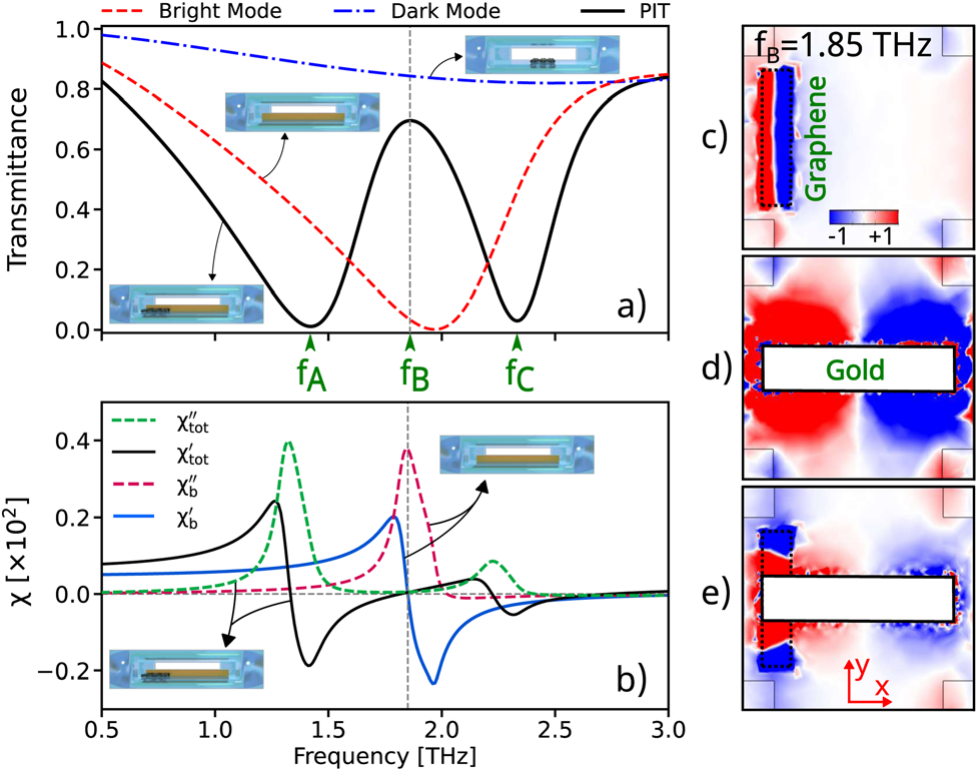
(a) The transmission spectra of the dark mode (the blue dashed-dot line), bright mode (the red dashed line), and PIT (the black solid line). The frequencies of two dips are located at *f*_A_ = 1.42 THz and *f*_c_ = 2.32 THz, while the peak appears at *f*_b_ = 1.85 THz. (b) The calculated real and imaginary parts of the susceptibility (eqn (6)–(8)). The green and red dashed lines, and the blue and black solid lines demonstrate the imaginary part and the real part, respectively. The electric field distributions 
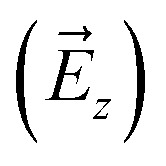
 corresponding to the (c) dark mode (graphene), (d) bright mode (gold), and (e) both of them.

Fig. 2(a) demonstrates the transmission spectra of the structure when the graphene strips are positioned at the end of the gold bars *s* = 22 μm and the Fermi level is set to *E*_F_ = 1 eV. This simulation, conducted using COMSOL Multiphysics, clearly illustrates the coupling between the dark and bright modes when the refractive index inside the pipe is *n* = 1.33. This adjustment of the Fermi level to 1 eV is achieved through electrostatic gating, representing a substantial electrostatic doping level achievable *via* external gate voltage.^43^

At a Fermi level of 1 eV, the plasmonic resonance frequency of graphene aligns well with the plasmonic resonance of gold and increases the carrier density in graphene. This alignment and higher carrier density enhance the coupling between the dark mode (graphene) and the bright mode (gold), leading to stronger plasmonic oscillations. These oscillations increase the electric field around the graphene strips, resulting in improved coupling with the gold bars. Fig. 2(b) illustrates the variation of the real and imaginary parts of the susceptibility of the structure under incident light. The figure clearly demonstrates that when *E*_F_ = 1 eV, the resonance becomes significantly more pronounced, indicating a heightened coupling between the gold and graphene components.

The real part of the susceptibility represents phase changes, while the imaginary part indicates the absorption or energy loss of the electric field within the structure. The excitation of the bright and dark modes causes peaks and dips in the imaginary part of the susceptibility, respectively. The imaginary part of the susceptibility shows significant variations at specific frequencies (close to the resonant frequencies of the dark and bright modes). These variations are due to the constructive and destructive interference between the dark and bright modes, leading to the PIT phenomenon.

The relative position of the gold and graphene strips leads to changes in the coupling, resulting in the formation of these peaks and dips in the imaginary part of the susceptibility. Therefore, when the graphene strip is located close to the edge of the gold bar and *E*_F_ = 1 eV, a stronger resonance and greater coupling between these modes occur, leading to a larger first peak. In contrast, at the second frequency, the peak is smaller due to greater destructive interference, demonstrating that the stronger resonance coupling occurs at *f*_A_.

The electric fields around the graphene and gold bars were obtained outside these materials, as shown in Fig. 2(c–e). Fig. 2(c) shows the electric field distribution around the graphene near the edges of the gold bars, in the absence of gold. The graphene strip (dark mode) cannot be directly excited by the incident light. This non-coupling is due to the symmetry and charge distribution on the graphene surface, which prevents direct excitation by the incident light. In contrast, the electric field is more widely distributed around the edges of the gold bar. This enhanced localization of surface plasmons at the sharp edges leads to higher field intensity, as shown in Fig. 2(d). As the graphene layer approaches the edges of the gold, the indirect coupling between the dark and bright modes is enhanced, as shown in Fig. 2(e).

**Fig. 3:**
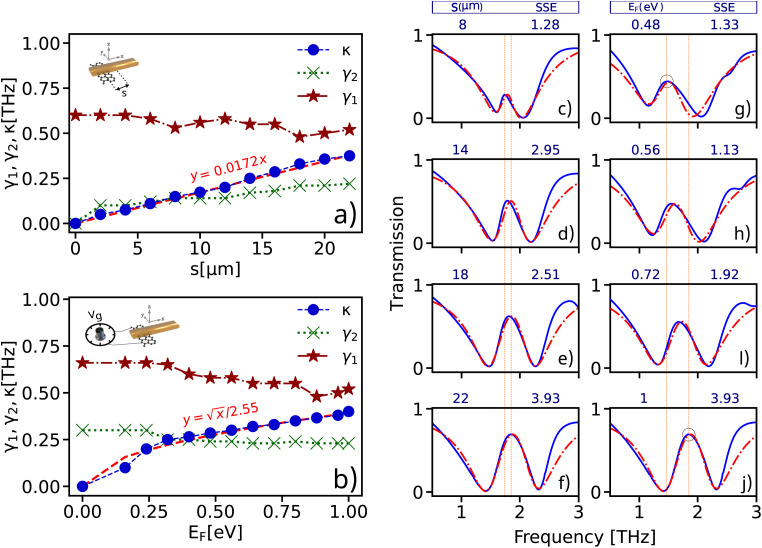
Extracted oscillator model parameters as functions of (a) the displacement (*s*) at *E*_F_ = 1 eV and, (b) the Fermi level of the graphene deposited under the gold at *s* = 22 μm. The fitting of the analytical oscillator model and numerical results, where red-dashed and blue solid line represent the analytical and numerical results, respectively. In figures (c–f) the Fermi level is set to *E*_F_ = 1 eV. The specific conditions and corresponding Sum of Squared Errors (SSE) are as follows: (c) *s* = 8 μm, SSE = 1.28, (d) *s* = 14 μm, SSE = 2.95, (e) *s* = 18 μm, SSE = 2.51, (f) *s* = 22 μm, SSE = 3.93. Also in figures (g–j) the displacement parameter is set to *s* = 22 μm and the Fermi level varies for (g) *E*_F_ = 0.48 eV, SSE = 1.33, (h) *E*_F_ = 0.56 eV, SSE = 1.33, (i) *E*_F_ = 0.72 eV, SSE = 1.92, (j) *E*_F_ = 1 eV, SSE = 3.93.

The Fig. 3 illustrates the extracted oscillator model parameters as functions of displacement (*s*) and the Fermi level *E*_F_ of graphene deposited under gold at specific conditions, as well as the fitting model used to prove the behavior of the structure. The Fig. 3(a) shows how the parameters of the oscillator model change as a function of displacement (*s*) when the Fermi level is set to *E*_F_ = 1 eV. The damping rate of the bright mode (*γ*_1_) and the coupling strength of the bright mode (*g*) with the incident light (eqn (5)) remain nearly constant (≈0.6 THz) as *s* varies, because gold, as a noble metal, has stable optical and electrical properties that are not affected by changes in the relative displacement of graphene.

The decay rate (*γ*_1_) is related to the intrinsic characteristics of gold, such as free electrons and plasma frequency, which are not influenced by these scales of displacement changes. The coupling strength (*g*) arises from the direct interaction of light with the gold bars, which is more dependent on the structural and material properties of gold than on the position of graphene. This interaction, due to phenomena like surface plasmon resonance in gold, remains relatively constant. The decay rate and coupling of the bright mode are more influenced by external parameters such as the frequency of light and the applied electric field rather than small changes in the position of graphene.

The damping rate of the dark mode (*γ*_2_) decreases from 0.4 THz to 0.22 THz, indicating that the dark mode loses less energy over time, which reflects an increased efficiency in retaining plasmonic energy within the graphene. As *γ*_2_ decreases, the coupling between the dark mode (graphene) and the bright mode (gold) improves. This is because a lower damping rate allows the dark mode to interact more effectively with the bright mode, receiving more energy from it. On the other hand, the reduction in *γ*_2_ implies that the dark mode can remain in an excited state for a longer duration. This extended lifetime of the dark mode enhances the stability of plasmonic oscillations, thereby increasing the structure's sensitivity and overall performance.

Additionally, the coupling coefficient *K* increasing from 0.012 THz to 0.375 THz that indicates a significant enhancement in the interaction strength between the modes. To illustrate the increase in this coefficient, an approximation for *K* is shown in red, indicating a positive slope for this parameter. This demonstrates that the coupling coefficient *K* nearly linearly increases with the spatial separation parameter *s* indicating a direct correlation between the geometric configuration and the interaction strength between the dark and bright modes.

Fig. 3(b) demonstrates how the parameters of the oscillator model change as a function of the Fermi level *E*_F_ of graphene when the displacement is fixed at *s* = 22 μm. As *E*_F_ rises from 0.16 eV to 0.24 eV, *K* increases from 0.1 THz to 0.22 THz, showing that higher Fermi level enhances the plasmonic activity of graphene, leading to stronger coupling with the gold bars. This behavior suggests that higher Fermi level enhances the plasmonic activity of graphene, leading to stronger coupling with the gold bars. Upon reaching approximately 0.4 THz, *K* saturates, indicating a threshold beyond which further increases in *E*_F_ do not significantly enhance coupling. This saturation point represents the maximum attainable coupling coefficient, considering variations in both the *s* parameter and *E*_F_.

The required parameters for describing the oscillator models for the bright and dark modes as functions of the displacement parameter (*s*) and the Fermi level *E*_F_ of the graphene are extracted by fitting the analytical models to the numerical result (see Fig. 3(c–j)) by using the mean least square method. The close alignment between the observed and predicted values indicates that the analytical model accurately describes the system, thereby confirming its validity and effectiveness in predicting the behavior of the plasmonic biosensor.

As shown in parts (c–f), with the increase in distance between the center of the dark and bright modes, a stronger interaction between the modes is formed. This leads to an increase in resonance amplitude because the dark mode can receive more energy from the bright mode, resulting in stronger plasmonic oscillations. Also, increasing *E*_F_ results in a significant frequency shift because the carrier density in graphene changes. However, the resonance amplitude decreases less because a higher Fermi level increases the concentration of electrons in graphene, which enhances graphene's ability to absorb and reflect plasmonic energy.

**Fig. 4:**
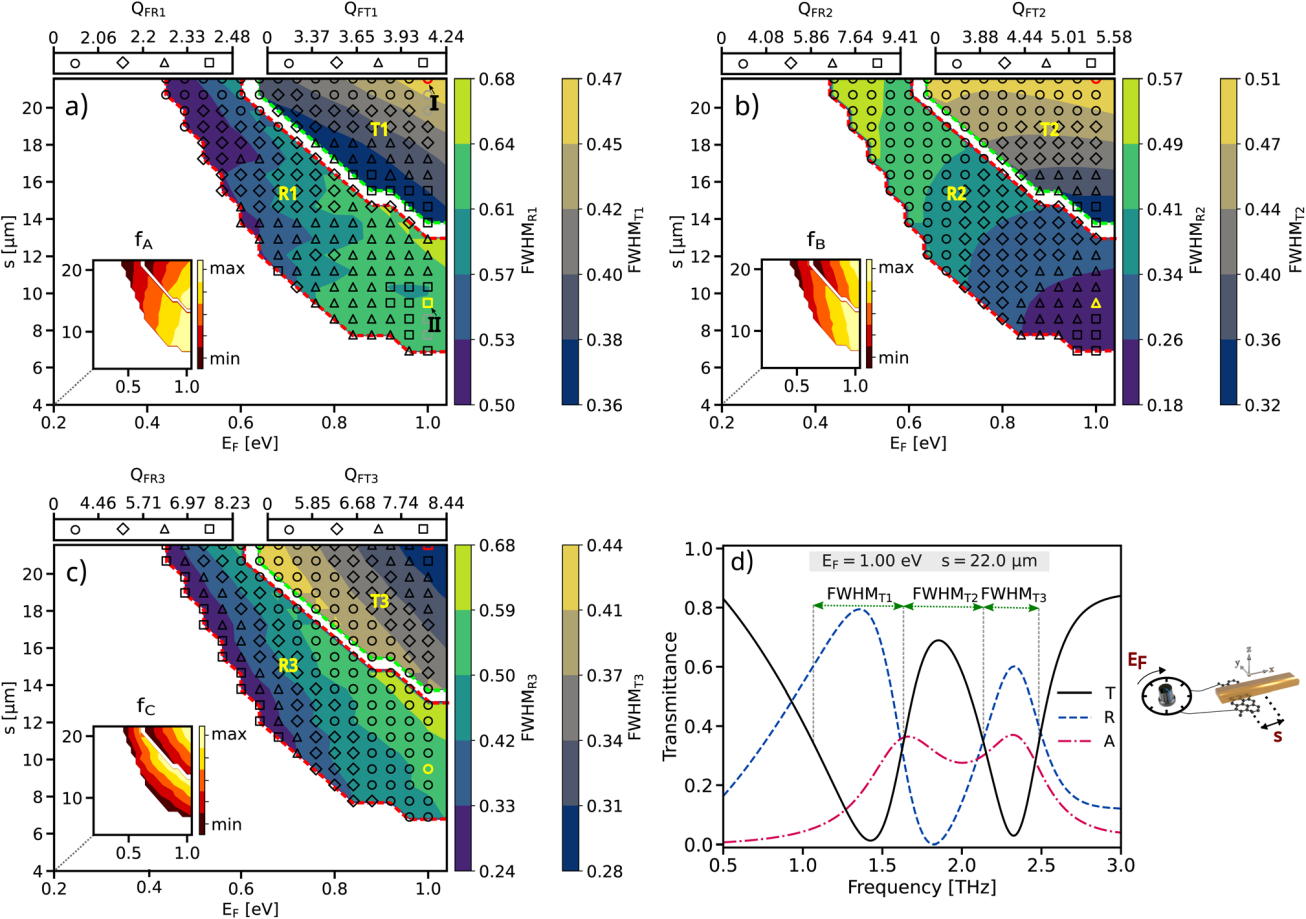
The FWHM, quality factor *Q*, and frequency values were analyzed for various pairs of parameters (*E*_F_, *s*) including: (a) the first dip *f*_A_, (b) the peak *f*_B_, and (c) the second dip *f*_C_. The FWHM is represented through color contours, and the *Q* values are denoted by different symbol – circles, diamonds, triangles, and squares – corresponding to increasing values, respectively. Further, the transmission, reflection, and absorption spectra characteristic – dips and peak – were examined for (d) *s* = 22 μm and *E*_F_ = 1 eV, and *s* = 11.2 μm and *E*_F_ = 1 eV.

To characterize the transmission and reflection spectra further, the full width at half maximum (FWHM) and the quality factor 
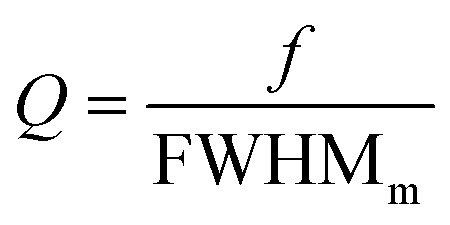
 were simulated using the COMSOL Multiphysics and analyzed in figure Fig. 4. According to the Lorentz model, when the graphene is positioned near the edges of the gold and the Fermi level is maintained at 1 eV, there is an increase in the coupling coefficient, dark mode damping rate, and FWHM.^44^ Across a broad range of (*E*_F_, *s*), numerous points with varying PIT peak values are identifiable. Points with PIT peak values below a certain threshold are considered negligible. The regions labeled *R*_1_, *R*_2_, and *R*_3_ indicate the conditions (all possible pairs of (*E*_F_, *s*)) where the transmission peak of the PIT is less than the reflection. Conversely, the regions marked *T*_1_, *T*_2_, and *T*_3_ show where the transmission peak exceeds 0.6 (60%).

Fig. 4(a) illustrates the FWHM and resonance frequency for the transmission and reflection spectrum at *f*_A_. Two points, I and II, represent the (*E*_F_, *s*) values where the FWHM and *f* are maximum in the transmission and reflection regions, respectively. According to (eqn (5)), when the amplitude of the imaginary part of susceptibility *χ*_*i*_ is higher, the value of *gχ*_*i*_ increasese. Based on Fig. 2(b), owing to the maximum value of the *χ*_*i*_ at *f*_A_, the transmission decreases.

Fig. 4(b) demonstrates a likely balanced in the FWHM in the transmission and reflection regions at *f*_B_. According to the Fig. 2(b), at this frequency, there is a symmetry in the real and imaginary parts of the susceptibility. This symmetry in imaginary part of susceptibility suggests that the energy interaction is evenly distributed between reflection and transmission. When the imaginary part (*χ*_*i*_) is balanced, it means the material neither heavily absorbs and reflects nor heavily transmits energy but does both to a similar extent.

Additionally, Fig. 4(c) shows that the FWHM of the transmission is higher than that of the reflection. Due to the value of *f*_C_, which is higher than *f*_A_ and *f*_B_, the quality factor is higher at this frequency. This higher quality factor at *f*_C_ indicates that the structure is more efficient in transmitting light, making it more suitable for biosensor applications where high transmission and sensitivity are crucial.

The frequency range of THz (0.1–10 THz) is particularly valuable for the detection of biomolecules such as proteins, DNA, viruses, and cancer cells.^14^ Previous work, such as that by Geng *et al.*,^14^ has demonstrated the efficacy of a THz biosensor embedded in microfluidics for liver cancer biomarker detection, highlighting how minimal sample volumes can yield precise detection results. Additionally, Zhang *et al.*'s EIT-based THz biosensor^23^ underscores the capability for detecting malignant glioma cells even at low concentrations. To investigate the biosensing capability of the proposed structure—particularly for detecting liver cancer biomarkers—the refractive index of the analyte inside the microfluidic channel was varied from 1.33 to 1.43 in steps of 0.01.^13^ Notably, a refractive index of 1.33 corresponds to the index of the deionized water used for pipe cleaning.^45^

**Fig. 5:**
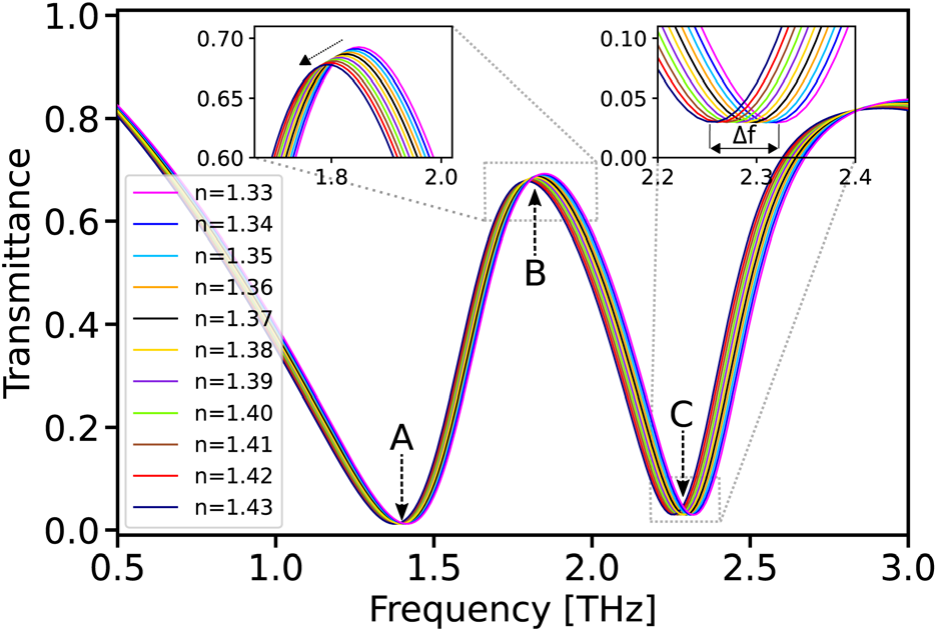
The sensitivities of the dip and peak frequencies are detailed as follows: the sensitivity analysis at the first dip (A), peak (B), and second dip (C) frequencies as a function of the refractive index.

The sensitivity of the device, defined as the ratio of the frequency shift to changes in the refractive index (*S* = Δ*f*/Δ*n*), depends on several factors, including the inverse FWHM of the incident light. For this purpose, the sensitivity of the structure at *E*_F_ = 1 eV and *s* = 22 μm was simulated, as shown in Fig. 5. The change in the refractive index of the analyte inside the pipe causes a change in the effective refractive index of the entire system, which can affect the resonance conditions and mode interference.

These changes can lead to variations in the transmission amplitude at different points of the spectrum. At points B and C, these changes appear differently due to specific interference conditions of the modes. At point B, it results in a decrease in amplitude, while at point C, it leads to the stability of the amplitude. The structure achieves a maximum sensitivity of 700 GHz per RIU at point C, demonstrating its capability to detect small variations in refractive index.

Compared to previously reported THz biosensors, the proposed system introduces several key innovations. First, it achieves a high sensitivity of approximately 700 GHz per RIU—significantly surpassing many existing designs—by leveraging hybrid plasmonic coupling between gold (bright mode) and graphene (dark mode), which enhances the PIT effect. Second, the integration of a microfluidic channel enables precise analyte delivery and minimizes environmental disturbances such as temperature fluctuations and vibrations, thereby improving stability and measurement reproducibility. Third, the system provides both static tunability—through geometric design during fabrication—and dynamic tunability *via* electrostatic gating of the graphene layer, allowing real-time control of the sensor's optical response. Finally, the use of graphene, with its strong light–matter interaction and high sensitivity to refractive index changes, further enhances detection capability beyond what is achievable with conventional metallic-only structures. These combined features position the proposed design as a versatile and high-performance platform for advanced THz biosensing applications. Table 1 provides a comparative analysis of several biosensors, highlighting the superior biosensing capabilities of the proposed structure.

**Table 1:** Comparative analysis of key characteristics of biosensors

Ref.	*n*_eff_	Frequency [THz]	Sensitivity [GHz per RIU]	Concept of the study
46	—	0.8–1.2	270.40	EIT metamaterials
14	1.33–1.40	0.4–1.0	150.00	Split-ring resonators
47	1.30–1.40	0.8–1.8	325.00	E-shaped resonators
48	1.60–2.00	0.15–0.85	139.20	Metamaterial absorber
49	1.00–2.00	0.4–0.6	126.00	Split-ring resonator
This study	1.33–1.43	0.5–3.0	700.00	PIT metamaterials

## Analytical model

To study the characteristics of the device, Maxwell's equations were numerically solved by using the finite element method (FEM) as implemented in the COMSOL Multiphysics software.^50^ Floquet periodic boundary conditions were applied to the lateral faces along the *x*- and *y*-directions to model an infinite 2D array of optical resonators. Perfectly matched layers (PML) were placed at the top and bottom boundaries to avoid reflections from the input and output ports, respectively. The frequency-dependent complex refractive indices of Au, based on the Drude model, was obtained from ref. 51:1
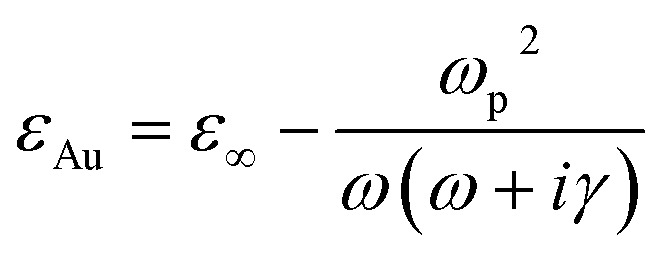
where *ε*_Au_ and *ε*_∞_ are the relative and high frequency gold permittivity, *ω*_p_ = 1.374 × 10^16^ Rad s^−1^ (ref. 51) is the plasma frequency and *γ* = 1.225 × 10^14^ Rad s^−1^ the damping constant.^51^ The refractive index of SiO_2_ is taken as *n*_SiO_2__ = 4.^52^

The frequency-dependent optical conductivity of graphene strips can be determined based on the random-phase approximation (RPA) that includes both intraband and interband transitions *σ*_Gr_ = *σ*_inter_ + *σ*_intera_.^53,54^ It is worth noting that nonlinear optical effects can arise from the strong local field enhancements at the graphene–metal interface, where the coupling of surface plasmons with the π-electrons of graphene may induce phenomena such as third-harmonic generation and Kerr-type nonlinearities.^55,56^ These effects originate from the intensity-dependent response of graphene's charge carriers under high field confinement. However, in this study, we have focused exclusively on the linear regime, using the Kubo formalism and moderate excitation intensities, where such nonlinear contributions are negligible. As such, nonlinear interactions are beyond the scope of the present model but may be considered in future investigations. However, interband transitions can be neglected at room temperature for sufficiently low for adequately low frequencies (ℏ*ω* ≤ 2*E*_F_).^57^ Intraband contributions of graphene conductivity have a Drude-like form in highly doped graphene stripes (*E*_F_ > *k*_B_*T*):^57,58^2
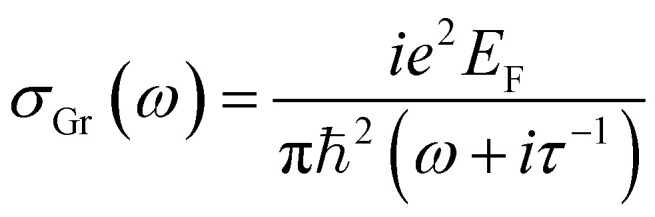
where *τ* = *μE*_F_/*eυ*_F_^2^ (ref. 51) is the carrier relaxation time, *μ* = 10 000 cm^2^ V^−1^ s^−1^ (ref. 52) is the carrier mobility, and *υ*_F_ = 1 × 10^6^ m s^−1^ is the Fermi velocity.^53^ A graphene sheet can be modelled as a thin dielectric layer with a permittivity *ε*_Gr_ that is given by:^59^3
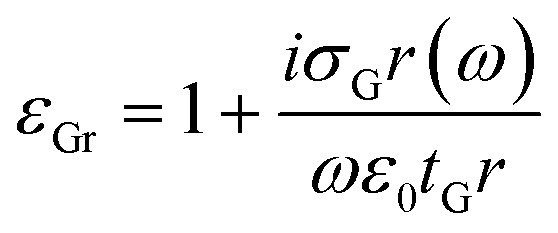
where *t*_Gr_ = 1 nm and *ε*_0_ are the graphene thickness and the vacuum permittivity, respectively. An analytical model of PIT effect parallel to numerical analysis is presented. The Lorentz oscillator model for two coupled oscillators can be utilized to analytically describe the interference between the modes.^60^ Based on this model the susceptibility *χ* is given by:^42,61^4
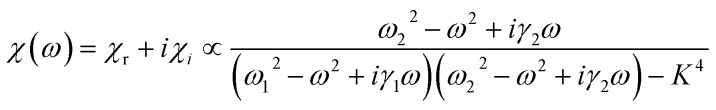
where the *ω*_1_ and *ω*_2_ are the natural oscillator frequencies, *γ*_1_ and *γ*_2_ are the damping rates, *K* is the coupling coefficient between the two resonance modes. In this study indices “1” and “2” represent Au bars and Gr strips, respectively. The transmission for *x*-polarized waves can be described by *T*(*ω*) = 1 − *gχ*_*i*_:^42^5
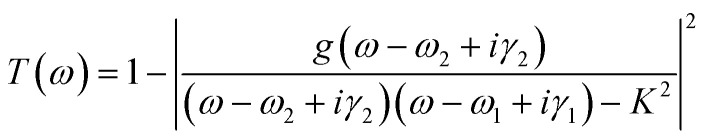


The elements of scattering matrix *S*_11_ and *S*_21_ will be evaluated from transfer matrix elements. The eqn (6) and (7) give the effective refractive index (*n*_r_) and impedance (*z*_r_) of the structure and eqn (8) completes the material description:^62,63^6
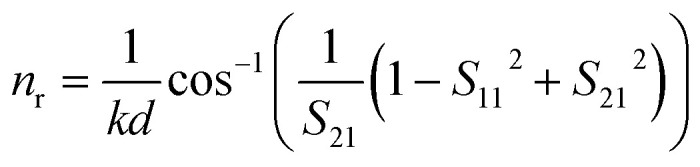
7
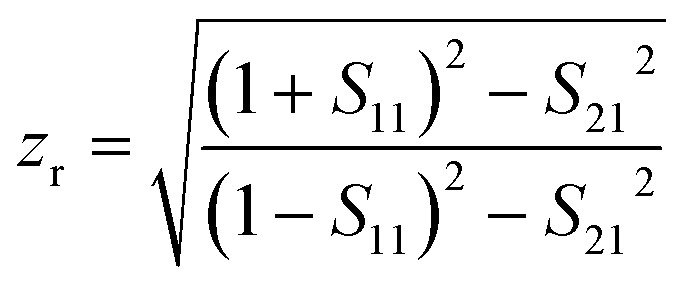
8
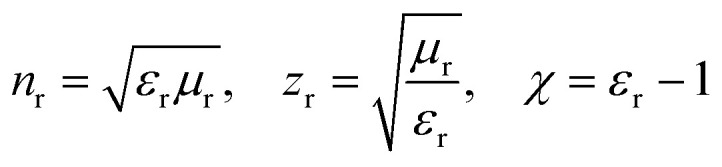


The analyte is modeled as a dielectric medium with a variable refractive index (1.33–1.43), uniformly filling the microfluidic channel where the plasmonic gold and graphene structures are located. Changes in the analyte's refractive index alter the local dielectric environment, thereby shifting the resonance frequency of the PIT response. This shift serves as the primary sensing mechanism. In simulations, the analyte's refractive index is assigned to the relevant dielectric domain, and the electromagnetic response is recalculated using FEM to extract sensitivity (Δ*f*/Δ*n*). This refractive index-based approach is standard for THz biosensors and does not assume molecular-level interactions.

## Conclusions

This work introduces a highly sensitive and tunable biosensor consisting of perpendicularly arranged graphene strips and gold bars. To mitigate environmental effects, all sensor modes are enclosed within pipes, with inlet and outlet channels enabling analyte refilling for enhanced reusability. Resonance frequencies and spectral domains can be precisely adjusted *via* electric field application and fabrication parameters. Numerical investigations and analytical models validate the results. Tunability is achieved by modifying the relative positions of graphene and gold components and by modulating the Fermi level. The biosensor shows a predicted sensitivity of 700 GHz per RIU for refractive index changes from 1.33 to 1.43, enabling detection of cancer cells, malaria infections, and glucose levels. Integration with microfluidics supports precise small-volume analysis, making it suitable for point-of-care and clinical use.^64^ Future work will focus on structural optimization for environmental robustness and the use of machine learning for multi-analyte detection and automated spectral interpretation.

## Author contributions

A. MF. conceived the idea, performed the simulations, and wrote the manuscript. M. P. discussed the results, reviewed the manuscript, and contributed to the scientific interpretation.

## Conflicts of interest

The authors declare that they have no known competing financial interests or personal relationships that could have appeared to influence the work reported in this paper.

## Supplementary Material

NA-007-D5NA00312A-s001

## Data Availability

The datasets supporting this article, including simulation data, raw results, have been archived and are available upon reasonable request from the corresponding author. Additionally, ESI† related to this study have been provided in the ESI† section of the manuscript. For further inquiries regarding data access, please contact the corresponding author at [pourfath@ut.ac.ir; pourfath@iue.tuwien.ac.at].
