# Supplementary material for: Sensitive terahertz plasmonic metasurface biosensor integrated with microfluidics
Source: Nanoscale Adv. 2025 Jun 12;7(15):4600–9. doi: 10.1039/d5na00312a (PMC12171736; doi:10.1039/d5na00312a)
Supplement: NA-007-D5NA00312A-s001 [file NA-007-D5NA00312A-s001.pdf]

Supplementary Information (SI) for Nanoscale Advances.
This journal is © The Royal Society of Chemistry 2025

Supporting Information

Sensitive Terahertz Plasmonic Metasurface Biosensor Integrated with Microfluidics

Amir Moradi Fotouhi[1,*] **and , Mahdi Pourfath**[1,2]

[1]School of Electrical and Computer Engineering, University of Tehran, Tehran, Iran
[2]Institute for Microelectronics, TU Wien, Gusshausstrasse 27-29/E360, 1040 Vienna, Austria.
[*]omid.fotouhi@ut.ac.ir
[+]mahdi.pourfath@tuwien.ac.at

S.1 Verification of the S-parameter model in an inhomogeneous medium

To validate the values obtained and presented in Figure 1(a) of the main text, the S-parameters from Smith et al. (2005)[1] and Chan et al. (2018)[3] were extracted using ANSYS and COMSOL Multiphysics software, respectively. Fig. 1(a) illustrates the 3D structure of the unit cell from Smith et al. (2005), the copper SRR (Split Ring Resonator) rings on one side of the surface and the copper wire on the opposite side were modeled as perfect electric conductors (*PEC*) based on the dimensions provided in the reference paper. Two air layers were placed on either side, each with equal height. The boundaries along the x-axis were defined as (*PEC*), while those along the z-axis were set as perfect magnetic conductors (*PMC*). Fig. 1(b) shows the magnitudes of the S-parameters (S_{11}) and (S_{21}), corresponding to the reflection and transmission of light from the structure, respectively

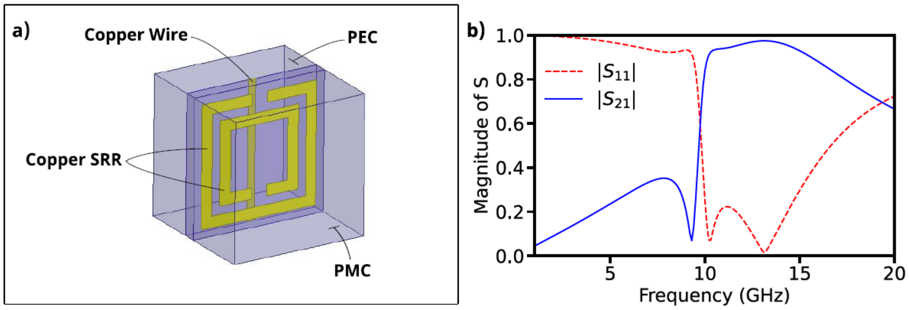

Figure 1. a) A single unit cell from Smith et al. (2005)[1] is shown. Two boundary conditions, PEC and PMC, are defined along the x and z directions, respectively. b) The magnitudes of S_{11} (red dashed line) and S_{21} (blue solid line) are shown.

Upon extracting the S-parameters from the reference model, these parameters were employed to validate Relations (6, 7, and 8) outlined in the main text of the article. The resulting optical parameters are depicted in Fig. 2.

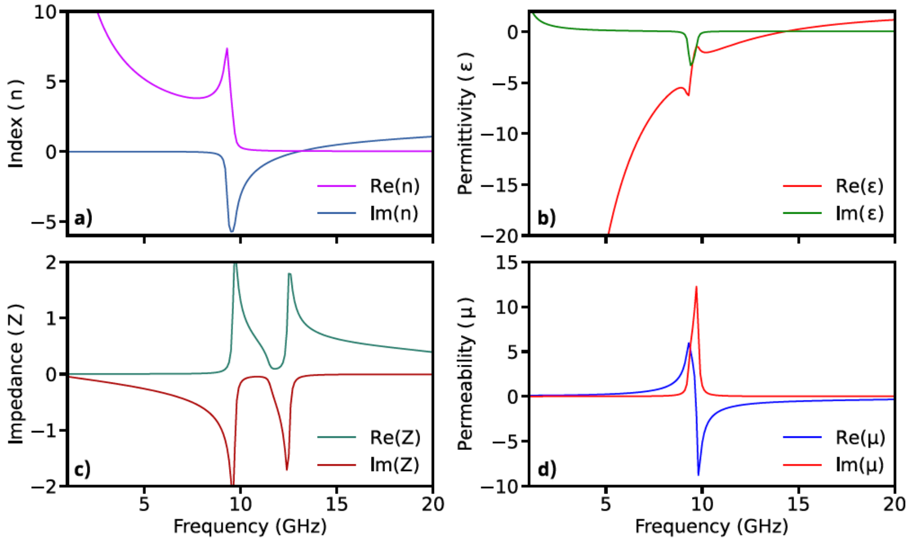

Figure 2. The optical parameters of the proposed structure, the real and imaginary parts of a) effective refractive index, b) the retrieved effective permittivity, c) Imedance, and d) effective permeability are shown.

To further substantiate the results presented in Figure 1 of the article, a simulation of the reference structure by Chan et al. (2018)[3] was performed using COMSOL Multiphysics. Fig. 3(a) illustrates the unit cell of the reference structure, with periodic boundary conditions (*PBC*) applied to the x and y planes. To model the permittivity of gold, Relation 3 from the article was utilized. The S-parameters were extracted, followed by the calculation of light transmission through the structure, as illustrated in Fig. 3(b).

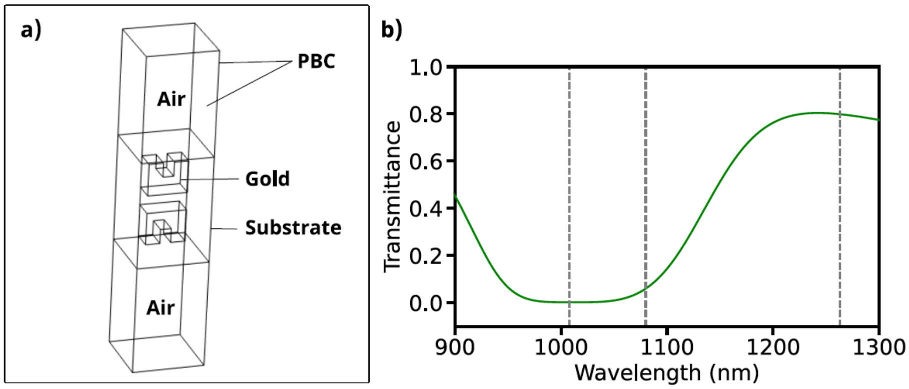

Figure 3. a) A single unit cell from Chan et al. (2018)[3] is shown. Periodic boundry condition are applied to x and y boundries. b) Spectra of transmittance of the proposed structure.

Considering that the thickness of gold in the first reference was negligible, perfect electric conductor (PEC) conditions were employed for modeling. However, since the thickness of the gold layer in the proposed structure shown in Figure 1 is significant, the S-parameters were recalculated to validate Relations 6, 7, and 8 for extracting the optical parameters in this context. The results are presented in Fig. 4.

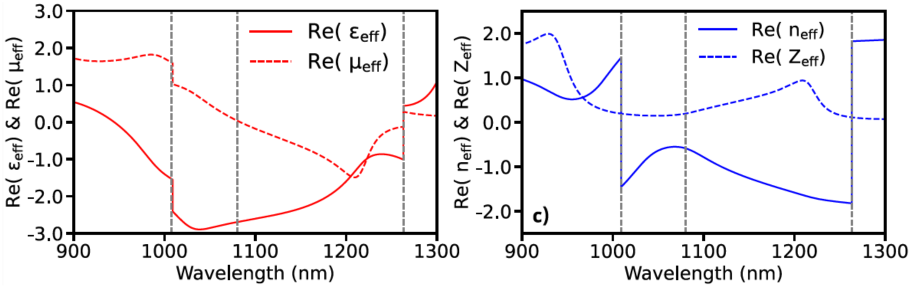

Figure 4. The optical parameters of the proposed structure are presented: a) the real part of the effective permittivity (red solid line) and effective permeability (red dashed line); and b) the effective refractive index (blue solid line) and effective impedance (blue dashed line).

To model the single-layer graphene in the structure presented in Sun et al. (2018)[2], the approach described by relation (2) in the main text was employed. Periodic boundary conditions were applied to each layer separately, and Transition boundary conditions were used for each graphene layer. The resulting absorption of the structure is illustrated in Fig. 5.

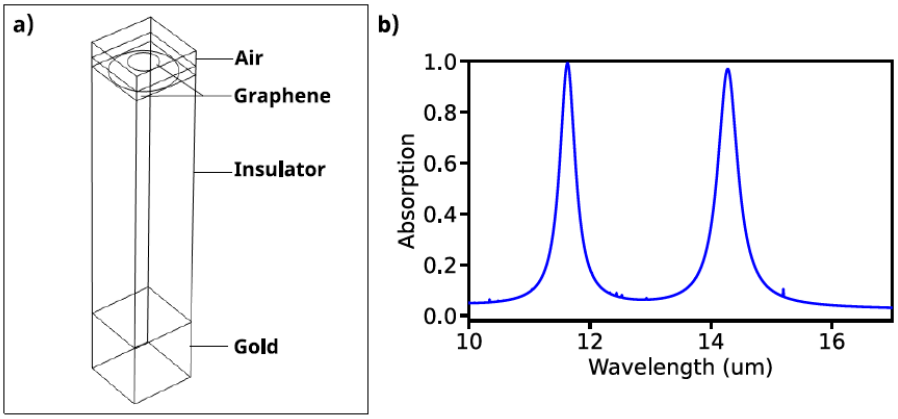

Figure 5. a) A single unit cell from Chan et al. (2018)[2] is shown. Periodic boundry condition are applied to x and y boundries for each layer. b) Spectra of transmittance of the proposed structure.

S.2 Initial Development of the Design via Circuit Modeling

The proposed structure was initially conceptualized and designed using an equivalent circuit model[4]. This approach systematically represents the plasmonic behavior of materials like gold and graphene. In this model, the inductance (L) reflects the energy stored in the magnetic field generated by oscillating electrons, which is strongly influenced by the geometry and material properties of gold and graphene[5]. Similarly, the capacitance (C) accounts for the energy stored in the electric field due to charge accumulation on the material surfaces, which is a function of the overlap area between the layers and the separation distance[10]. The resistance (R) models the energy dissipation through electron scattering and damping of oscillations, which is directly related to the intrinsic conductivity and relaxation times of the materials[7]. The coupling between the bright (gold) and dark (graphene) modes, arising primarily due to their spatial proximity and electromagnetic interactions, was modeled as a series capacitance in the equivalent circuit[8]. This coupling capacitance, determined by the overlap area and separation distance, increases as the distance decreases, thereby enhancing the interaction strength between the modes[9]. The dimensions of the gold and graphene layers, assumed to be rectangular, play a significant role in determining the equivalent circuit parameters, including inductance (L), resistance (R), and capacitance (C). These parameters are critical in modeling the plasmonic behavior of the bright (gold) and dark (graphene) modes.

S.2.1 Bright Mode (Gold)

The inductance L_b of the bright mode is governed by the geometry of the gold layer and can be expressed as:

$$L_b = \frac{\mu_0 L_{Au}}{W_{Au}} \tag{1}$$

Where μ_0 is the vacuum permeability, L_{Au} is the length, and W_{Au} is the width of the gold layer[5]. The resistance R_b relates to the gold layer's intrinsic resistivity and dimensions:

$$R_b = \frac{\rho_{Au} L_{Au}}{W_{Au} t_{Au}} \tag{2}$$

Where ρ_{Au} is the resistivity of gold, and t_{Au} is its thickness. This parameter represents energy dissipation due to material losses[10]. The capacitance C_b is influenced by the area of the gold layer and the separation distance (d) to the graphene:

$$C_b = \frac{\varepsilon_0 L_{Au} W_{Au}}{d} \tag{3}$$

Where ε_0 is the vacuum permittivity. This capacitance accounts for the electric field interaction between the gold and graphene layers[11].

S.2.2 Dark Mode (Graphene)

The inductance L_d of the dark mode depends on the geometry of the graphene layer:

$$L_d = \frac{\mu_0 L_{Gr}}{W_{Gr}} \tag{4}$$

Where L_{Gr} is the length, and W_{Gr} is the width of the graphene layer. The inductance captures the magnetic energy storage in the graphene plasmon mode[5]. The resistance R_d is defined by the intrinsic resistivity and dimensions of the graphene layer:

$$R_d = \frac{\rho_{Gr} L_{Gr}}{W_{Gr} t_{Gr}} \tag{5}$$

Where ρ_{Gr} is the resistivity of graphene, and t_{Gr} is its thickness. This parameter describes energy loss in the dark mode due to electron scattering[10]. The capacitance C_d is determined by the graphene layer's dimensions and its separation distance (d) from the gold:

$$C_d = \frac{\varepsilon_0 L_{Gr} W_{Gr}}{d} \tag{6}$$

This capacitance reflects charge accumulation and energy storage in the graphene's electric field[11].

Using this equivalent circuit framework, depicted in Fig. 6, we derived key parameters such as resonance frequencies, damping factors, and the plasmon-induced transparency (PIT) peak, aligning them with the operational frequencies shown in Figure 2 of the manuscript. This robust modeling approach also enabled iterative optimization of the physical dimensions of the gold and graphene layers to enhance the PIT effect and fine-tune the dynamic coupling between the modes for optimal performance.

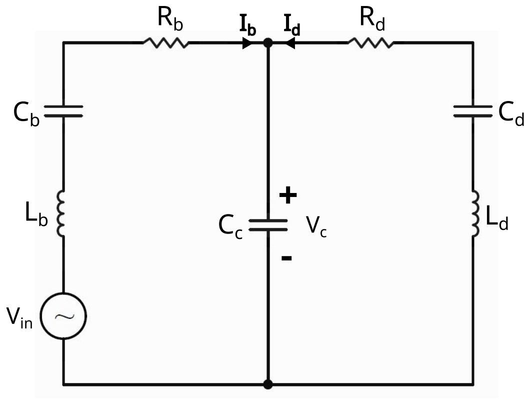

Figure 6. The circuit model represents the system's operation, where L_b, C_b, and R_b correspond to the inductance, capacitance, and resistance of the bright mode, while L_d, C_d, and R_d represent the same elements for the dark mode. The coupling capacitance is denoted by C_c, and the variable voltage source V_{in} provides the input excitation to the system.

S.2.3 Coupling Between Modes

The coupling between the bright and dark modes is modeled as a coupling capacitance C_c:

$$C_c = \frac{\varepsilon_0 A_{\text{Overlap}}}{d} \tag{7}$$

Where $A_{\text{Overlap}} = \min(L_{\text{Au}}, L_{\text{Gr}}) \cdot \min(W_{\text{Au}}, W_{\text{Gr}})$ is the overlap area between the gold and graphene layers, and (d) is the separation distance. This capacitance quantifies the electric field interaction between the two modes and governs their coupling strength[9].

S.2.4 Circuit analysis:

For bright mode loop:

$$V_c + \frac{1}{j\omega C_b} I_b + j\omega L_b I_b + R_b I_b = V_{in} \tag{8}$$

For dark mode loop:

$$\frac{1}{j\omega C_d} I_d + j\omega L_d I_d + R_d I_d = V_c \tag{9}$$

Calculating the value of V_c:

$$V_c = \frac{1}{j\omega C_c}(I_b - I_d) \tag{10}$$

We substitute the value of V_c into equations 8 and 9, and then simplify the expressions.

$$\frac{1}{j\omega C_c}(I_b - I_d) + \frac{1}{j\omega C_b} I_b + j\omega L_b I_b + R_b I_b = V_{in} \tag{11}$$

$$\frac{1}{j\omega C_d} I_d + j\omega L_d I_d + R_d I_d = \frac{1}{j\omega C_c}(I_b - I_d) \tag{12}$$

Conventionally, Z_b and Z_d are defined as follows:

$$Z_b = \frac{1}{j\omega C_b} + j\omega L_b + R_b \tag{13}$$

$$Z_d = \frac{1}{j\omega C_d} + j\omega L_d + R_d \tag{14}$$

Therefore, equations 11 and 12 can be simplified as follows:

$$\frac{1}{j\omega C_c}(I_b - I_d) + Z_b I_b = V_{in} \tag{15}$$

$$\frac{1}{j\omega C_c}(I_b - I_d) = Z_d I_d \tag{16}$$

Since the susceptibility is calculated from the ratio of the bright-mode current to the input voltage, we have:

$$I_b = \frac{V_{in}}{\frac{1}{Z_d} + \frac{1}{j\omega C_c} + Z_b} \tag{17}$$

$$\chi(\omega) = \frac{I_b}{V_{in}} \tag{18}$$

$$\chi(\omega) = \frac{1}{\frac{1}{Z_d} + \frac{1}{j\omega C_c} + Z_b} \tag{19}$$

Considering the resonance frequency, damping coefficients, and coupling coefficient for each mode, the susceptibility can be determined as follows. Consequently, the light transmission of the structure can be derived from it:

$$\begin{cases} \omega_1^2 = \frac{1}{C_b L_b} \\ \omega_2^2 = \frac{1}{C_d L_d} \\ \gamma_1 = \frac{R_b}{L_b} \\ \gamma_2 = \frac{R_d}{L_d} \\ K = \frac{1}{C_c} \end{cases} \tag{20}$$

$$Z_b \to i\gamma_1 \omega + \omega^2 - \omega_1^2 \tag{21}$$

$$Z_d \to i\gamma_2 \omega + \omega^2 - \omega_2^2 \tag{22}$$

$$K^2 \to \frac{1}{j\omega C_c} \tag{23}$$

$$\chi(\omega) = \chi_r + i\chi_i \propto \frac{{\omega_2}^2 - \omega^2 + i\gamma_2\omega}{({\omega_1}^2 - \omega^2 + i\gamma_1\omega)({\omega_2}^2 - \omega^2 + i\gamma_2\omega) - K^4} \tag{24}$$

$$T(\omega) = 1 - g\chi_i \tag{25}$$

To design a structure with resonance frequencies in the range of $0.5THz$ to $3THz$, we follow a step-by-step approach. The calculations ensure that the geometric dimensions of gold and graphene approximate those in the article while achieving the desired frequency range.

S.2.5 Resonance Frequency f_r:

$$f_r = \frac{1}{2\pi\sqrt{LC}} \tag{26}$$

To achieve resonance frequencies within the range $0.5\,\mathrm{THz} \leq f_r \leq 3\,\mathrm{THz}$, L and C must be appropriately tuned based on the geometry and material properties of gold and graphene.

$$\frac{1}{(2\pi f_{\max})^2} \leq LC \leq \frac{1}{(2\pi f_{\min})^2} \tag{27}$$

Using $f_{\min} = 0.5\,\mathrm{THz} = 0.5 \times 10^{12}\,\mathrm{Hz}$ and $f_{\max} = 3\,\mathrm{THz} = 3 \times 10^{12}\,\mathrm{Hz}$, the range for L and C is:

$$L.C \in \left[2.81 \times 10^{-27}, 1.01 \times 10^{-25}\right] \mathrm{H} \cdot \mathrm{F} \tag{28}$$

S.2.6 Determining Layer Dimensions:

We evaluated various dimensions (lengths and widths) for the bright (gold) and dark (graphene) modes by substituting them into equations 1 through 7. The calculations of inductance (L) and capacitance (C) allowed us to identify the optimal configuration that satisfies the constraints in equation 28. Using the dimensions provided in the article ($L_{\mathrm{Au}} = 50\,\mu$m, $W_{\mathrm{Au}} = 12\,\mu$m, $L_{\mathrm{Gr}} = 35\,\mu$m, $W_{\mathrm{Gr}} = 6\,\mu$m), it was observed that these values are specifically tuned for the bright and dark modes to achieve resonance frequencies optimized for PIT. The higher capacitance and appropriately adjusted inductance in these dimensions create the strongest coupling, leading to a pronounced PIT peak. This configuration ensures maximum energy transfer and interference between the modes, resulting in the most significant PIT effect within the operational range of 0.5 to 3 THz. After initially estimating the dimensions using the analytical model, we utilized FEM simulations in COMSOL Multiphysics to refine the design and validate the results. These simulations enabled us to systematically investigate various geometric configurations and their effects on the PIT peak within the target frequency range of 0.5 to 3 THz. By iterating over different values for the length, width, and separation distance, we identified the optimal configuration that maximizes the coupling between the bright and dark modes, leading to the most pronounced PIT peak. The Fig. 7 illustrates the simulation results, demonstrating the optimized dimensions and their corresponding PIT performance.

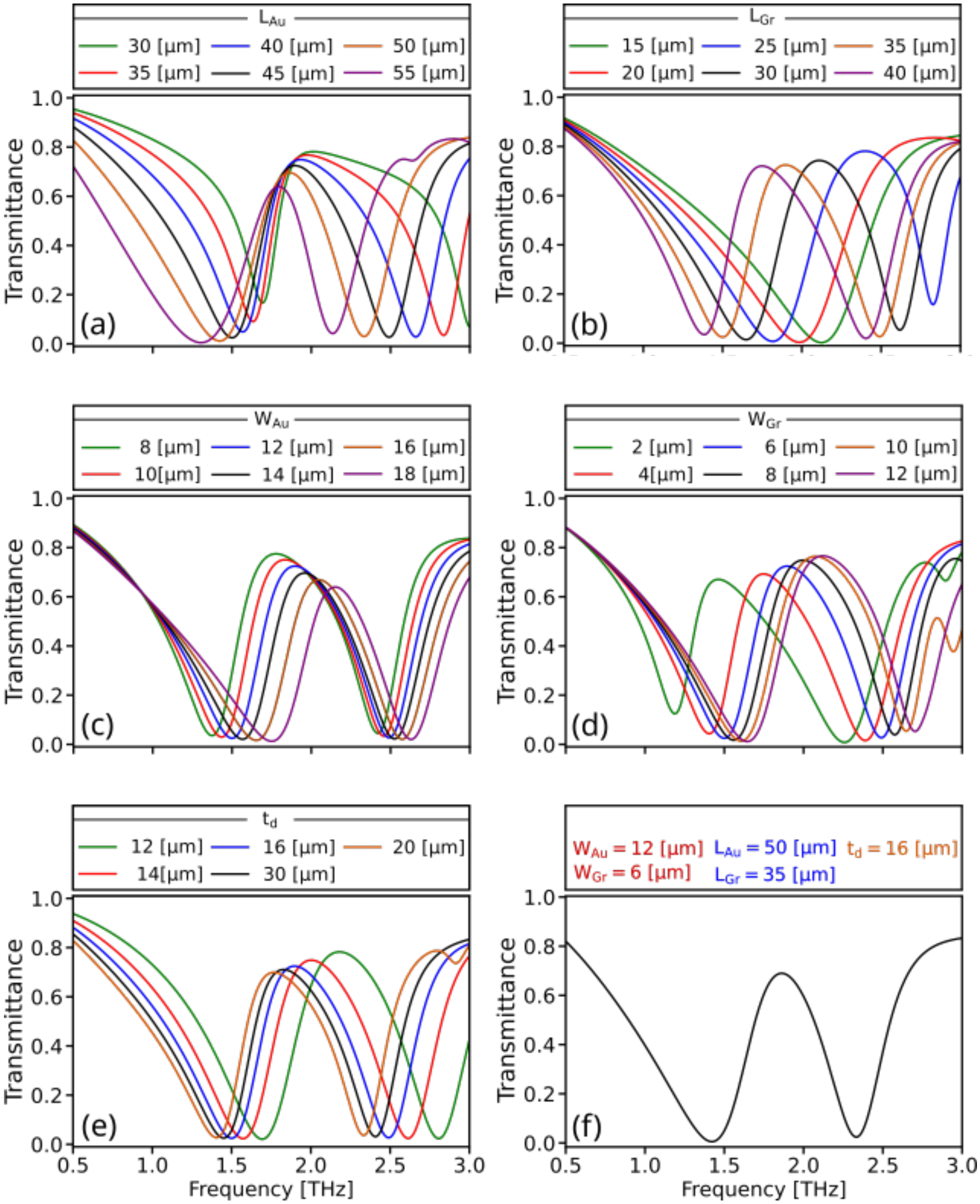

Figure 7. Transmission spectra of the structure calculated for variations in different parameters: (a) L_{Au}, (b) L_{Gr}, (c) W_{Au}, (d) W_{Gr}, (e) tube thickness, and (f) optimized parameter values.

S.2.7 Impact of Fermi Energy Variation on the PIT Response:

In Fig. 8(a), we study the impact of varying the separation distance (s) between the gold and graphene elements at a fixed Fermi energy of $E_F = 1eV$. As s increases from 8.6 to $s = 22\mu m$, the coupling strength between the bright and dark modes decreases significantly due to the weakened near-field interaction. This reduction in coupling strength results in a notable decrease in the amplitude of the PIT window. However, since the intrinsic resonance frequencies of the bright and dark modes remain largely unchanged, the central frequency of the PIT feature experiences only a slight shift. In contrast, Fig. 8(b) illustrates the effect of varying the Fermi energy E_F from 1 eV down to 0.6 eV while keeping s fixed at $s = 22\,\mu m$. As E_F decreases, the plasmonic resonance frequency of the graphene strip shifts to lower values, consistent with the relation $\omega_p \propto \sqrt{E_F}$. This results in a significant redshift of the PIT window. Although the reduction in E_F slightly diminishes the plasmonic strength of the dark mode—and thus the coupling strength—the corresponding decrease in PIT amplitude is relatively modest compared to the s-dependent case. Therefore, variation in E_F primarily controls the spectral position of the PIT window, whereas variation in s predominantly modulates its amplitude and visibility.

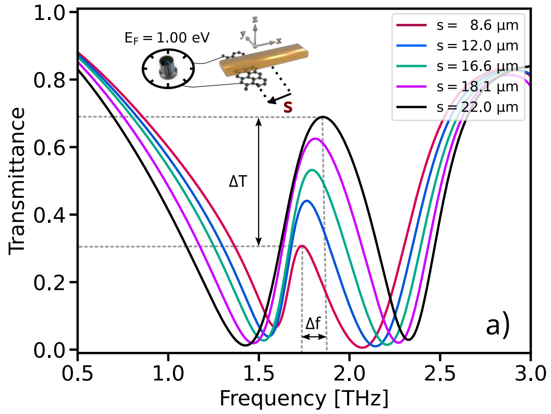

Figure 8. (a) Transmission spectra for different distances s between the gold and graphene elements at a fixed Fermi energy of $E_F = 1eV$. As s increases from $s = 8.6\mu m$ to $s = 22\mu m$, the amplitude of the PIT window decreases significantly due to reduced near-field coupling, while the resonance frequency exhibits only minor shifts. (b) Transmission spectra for different Fermi energies E_F from 1 eV down to 0.6 eV at a fixed distance $s = 22\mu m$. As E_F decreases, the PIT resonance undergoes a pronounced red-shift due to the change in the plasmonic resonance of graphene, while the PIT amplitude shows a relatively moderate reduction.

S.2.8 Figure of merit:

Fig. 9(a–c) illustrates the sensitivity of the three key resonance frequencies (dips A and C, and PIT peak B). The sensitivity is highest at point C, followed by B, and then A. This trend is attributed to the narrower spectral linewidth at point C, resulting in a higher Q-factor and, consequently, a larger frequency shift (Δf) for a given refractive index change, which defines the sensitivity (S). Thus, S_C is the largest among the three points. Fig. 9(d–f) shows that the FOM at point C (FOM_C) is also the highest, since $FOM = S/\text{FWHM}$[12], and both a large S_C and a small FWHM_C are observed. Conversely, at point A (the first dip), a broader linewidth and lower Q-factor result in a smaller Δf and S_A, leading to the lowest FOMA. The PIT peak (point B) shows intermediate performance. In summary, Fig. 9 demonstrates that regions with narrower spectral features (e.g., point C) exhibit superior sensitivity and FOM, whereas broader spectral features (e.g., point A) correspond to reduced sensing performance. Importantly, while high sensitivity contributes positively to the FOM, an overly broad linewidth can significantly reduce it. This trade-off arises from the interplay between coupling strength and spectral resolution: strong coupling increases Δf but may also broaden the linewidth, lowering the FOM. From a design standpoint, tuning structural parameters like gap (s) and Fermi energy (E_F) enables optimization of both sensitivity and FOM. As shown in Figure 3 of the main manuscript, increasing s enhances coupling but also broadens the spectral response. Therefore, optimal sensor performance requires balancing these effects to simultaneously maximize energy transfer and maintain narrow spectral features for high Q and FOM.

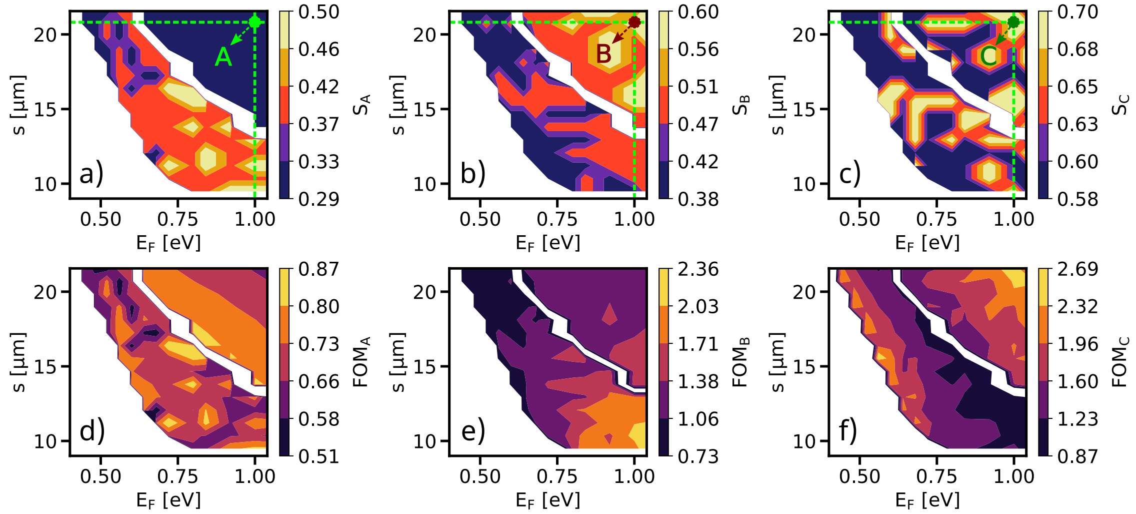

Figure 9. The sensitivities of the dips and peak frequencies, a) S_A, b) S_B, and c) S_C). The FOMs of dip and peak frequencies d) FOM_A, e)FOM_B, and f)FOM_C. For all calculations $E_F = 1eV$ and $s = 12\mu m$.

References

1. D. R. Smith, D. C. Vier, T. Koschny, and C. M. Soukoulis, "Electromagnetic parameter retrieval from inhomogeneous metamaterials," Phys. Rev. E-Statistical, Nonlinear, Soft Matter Phys. **71**, 036617 (2005).
2. P. Sun, C. You, A. Mahigir, T. Liu, F. Xia, W. Kong, G. Veronis, J. P. Dowling, L. Dong, and M. Yun, "Graphene-based dual-band independently tunable infrared absorber," Nanoscale **10**, 15564–15570 (2018).
3. H.-C. Chan, S. Sun, and G.-Y. Guo, "Near-infrared left-handed metamaterials made of arrays of upright split-ring pairs," J. Phys. D: Appl. Phys. **51**, 265103 (2018).
4. K. M. Devi, A. K. Sarma, D. R. Chowdhury, and G. Kumar, "Plasmon induced transparency effect through alternately coupled resonators in terahertz metamaterial," Opt. Express **25**, 10484–10493 (2017).
5. A. N. Grigorenko, M. Polini, and K. S. Novoselov, "Graphene plasmonics," Nat. Photonics **6**, 749–758 (2012).
6. G. W. Hanson, "Dyadic Green's functions and guided surface waves for a surface conductivity model of graphene," J. Appl. Phys. **103**, 6 (2008).
7. F. H. L. Koppens, D. E. Chang, and F. J. García de Abajo, "Graphene plasmonics: a platform for strong light–matter interactions," Nano Lett. **11**, 3370–3377 (2011).
8. J. Zhang, N. Mu, L. Liu, J. Xie, H. Feng, J. Yao, T. Chen, and W. Zhu, "Highly sensitive detection of malignant glioma cells using metamaterial-inspired THz biosensor based on electromagnetically induced transparency," Biosens. Bioelectron. **185**, 113241 (2021).
9. X. Zhao, C. Yuan, L. Zhu, and J. Yao, "Graphene-based tunable terahertz plasmon-induced transparency metamaterial," Nanoscale **8**, 15273–15280 (2016).
10. G. W. Hanson, "Dyadic Green's functions and guided surface waves for a surface conductivity model of graphene," J. Appl. Phys. **103**, 6 (2008).
11. X. Zhao, C. Yuan, W. Lv, S. Xu, and J. Yao, "Plasmon-induced transparency in metamaterial based on graphene and split-ring resonators," IEEE Photonics Technol. Lett. **27**, 1321–1324 (2015).
12. B. Sensale-Rodriguez, R. Yan, M. M. Kelly, T. Fang, K. Tahy, W. S. Hwang, D. Jena, L. Liu, and H. G. Xing, "Broadband graphene terahertz modulators enabled by intraband transitions," Nat. communications **3**, 780 (2012).
